# Supplementary material for: A pilot randomized trial of incentive strategies to promote HIV retesting in rural Uganda
Source: PLoS One. 2020 May 29;15(5):e0233600. doi: 10.1371/journal.pone.0233600 (PMC7259772; doi:10.1371/journal.pone.0233600)
Supplement: S1 File — (PDF) [file pone.0233600.s003.pdf]

IBIS-Health Study - Aim 3 Pilot
Baseline questionnaire

Bracelet ID	Respondent name	
District Name	Parish Name	Village Name
Interviewer ID		
Date of Interview (DD/MM/YYYY)	Time of interview (HH:MM:SS)	
Recruitment venue:		

Section A. Demographic and socio-economic information

NO.	QUESTIONS	CODING CATEGORIES	RESPONSE
1	I'd like to begin by asking, what is your age?	NUMBER OF YEARS (2 DIGITS)<br><br>98 DON'T KNOW	
2	What is your mobile phone number?	MOBILE NUMBER (10 DIGITS)<br><br>0 IF NO MOBILE PHONE NUMBER	
3	Have you attended any school?	1 YES<br>2 NO →**A5**	
4	What is the highest level of school you have attended?	1 PRIMARY<br>2 O-LEVEL – INCOMPLETE<br>3 O-LEVEL – COMPLETE<br>4 A-LEVEL – INCOMPLETE<br>5 A-LEVEL – COMPLETE<br>6 TERTIARY SCHOOL<br>7 UNIVERSITY<br><br>98 DON'T KNOW	
5	What is your current marital status?	1 MARRIED, LIVING TOGETHER<br>2 MARRIED, NOT LIVING TOGETHER<br>3 NOT MARRIED, LIVING TOGETHER<br>4 DIVORCED/SEPARATED<br>5 WIDOWED<br>6 NEVER MARRIED AND NOT LIVING TOGETHER	
6	Do you have any biological children?	1 YES<br>2 NO	

7	Does **your household** have any of these items:<br><br>READ ALL OPTIONS<br><br>A. A clock or watch?<br><br>B. Electricity?<br><br>C. Radio?<br><br>D. Television?<br><br>E. Mobile telephone by any member?<br><br>F. Refrigerator?<br><br>G. Solar panel?<br><br>H. Bicycle?<br><br>I. Motorcycle?<br><br>J. Car or truck?	SELECT ALL THAT APPLY<br><br>YES NO<br>CLOCK/WATCH 1 2<br><br>ELECTRICITY 1 2<br><br>RADIO 1 2<br><br>TELEVISION 1 2<br><br>MOBILE PHONE 1 2<br><br>REFRIGERATOR 1 2<br><br>SOLAR PANEL 1 2<br><br>BICYCLE 1 2<br><br>MOTORCYCLE 1 2<br><br>CAR OR TRUCK 1 2	
8	How many of the following animals does your household own?<br><br>Cows?<br><br>Goats?<br><br>Sheep?<br><br>Chickens?	FOR EACH ANIMAL, ENTER NUMBER OWNED<br><br>COWS<br><br>GOATS<br><br>SHEEP<br><br>CHICKEN<br><br>0 NONE<br><br>97 IF MORE THAN 97<br>98 DON'T KNOW<br>99 REFUSE TO ANSWER	
9	How much would it cost you to travel to your preferred HIV testing venue?	ENTER NUMBER	

10	What is your primary occupation?	1 - Farmer who owns land<br>2 - Farmer who works on land owned by another<br>2 - Fishing/Fishmonger<br>3 - Shopkeeper/Market vendor<br>4 - Bar owner/Bar worker<br>5 - Transport [truck, taxi, motorcycle, bike, boat] drivers<br>6 - Hotel/Restaurant worker<br>7 - Tourism<br>8 - Teacher<br>9 - Student<br>10 - Government worker<br>11 – Military/Police/Security<br>12 - Housewife<br>13 - Household worker<br>14 - Healthcare worker<br>15 - Construction worker<br>16 - Factory worker<br>17 - Mining<br>18 - Commercial sex worker<br>19 – Office job/Secretary<br>20 - Disabled<br>21 - No job<br><br>99 - Other: ______________________<br>-7: Don't know	
11	How much money do you <u>normally</u> make in one week of doing work and other income generating activities? Please do not count money earned by your family, or money earned as interest or from renting goods and land to others.<br><br>*IF THE PARTICIPANT RESPONDS WITH A SALARY, DIVIDE BY THE NUMBER OF WORKING DAYS TO OBTAIN WEEKLY INCOME*	MONEY EARNED PER WEEK<br>(IN SHILLINGS)	
12	Where is your primary workplace?	1 OWN HOUSE/COMPOUND<br>2 THIS VILLAGE<br>3 NEIGHBORING VILLAGE<br>4 NEARBY MARKET CENTER<br>5 MBARARA TOWN<br>6 KAMPALA<br>7 ANOTHER DISTRICT<br>8 OTHER ________________<br>(SPECIFY)	
13	In the past <u>12 months</u>, about how many months in total have you been away from your home?	NUMBER OF MONTHS (2 digits)<br><br>98 DON'T KNOW	

Section B. Health and sexual behavior			
NO.	QUESTIONS	CODING CATEGORIES	RESPONSE
1	Have you felt sick or had any type of illness in the past 4 weeks?	1 YES<br>2 NO	
2	In the past 4 weeks, have you visited a dispensary or health facility in order to seek treatment or advice about your health?	1 YES<br>2 NO→**B4**	
3	Which health facility did you visit for health care?	1 HEALTH FACILITY LEVEL 4<br>2 HEALTH FACILITY LEVEL 3<br>3 HEALTH FACILITY LEVEL 2<br>4 VHT<br>5 DISPENSARY<br>6 TRADITIONAL HEALER<br>7 OTHER ______________<br>(SPECIFY)	
4	Have there been any days in the past 4 weeks when you felt too sick to be able to do any work?	1 YES<br>2 NO	
5	Have you smoked cigarettes at any time in the past 12 months?	1 YES<br>2 NO →**B8**	
6	Do you currently smoke cigarettes?	1 YES<br>2 NO →**B8**	
7	In the last 1-week, how many cigarettes did you smoke?	NUMBER OF CIGARETTES<br><br>98 DON'T KNOW	
8	Have you consumed alcoholic beverages at any time in the past 12 months?	1 YES<br>2 NO →**B10**	
9	How many drinks of alcohol (bottles, glasses, etc.) do you normally have in a week?	NUMBER OF DRINKS	
READ TO RESPONDENT: Now my next questions are about HIV and AIDS. I am first going to ask you questions about AIDS, and for each one I'd like for you to tell me your answer.			
10	Do you think your chances of currently having HIV/AIDS are high, moderate, low, or no risk at all?	1 HIGH →**B12**<br>2 MODERATE →**B12**<br>3 LOW<br>4 NO RISK AT ALL<br><br>98 DON'T KNOW →**B13**<br>99 REFUSED TO ANSWER →**B13**	
11	Why do you think that you have a <u>low</u> chance or <u>no risk</u> of having HIV/AIDS?<br><br>SELECT ALL MENTIONED; <u>DO NOT READ CHOICES</u>	1 IS NOT HAVING SEX<br>2 USES CONDOM<br>3 HAS ONLY ONE PARTNER<br>4 LIMITS THE NUMBER OF PARTNERS<br>5 PARTNER HAS NO OTHER PARTNERS<br>6 ALL PARTNERS ARE HIV NEGATIVE<br>7 HAS BEEN CIRCUMCISED<br>8 OTHER______________ (SPECIFY)<br><br>**SKIP TO B13 AFTER THIS**	
12	Why do you think that you have a <u>moderate</u> or <u>high chance</u> of having HIV/AIDS?<br><br>SELECT ALL MENTIONED; <u>DO NOT READ CHOICES</u>	1 DOES NOT USE CONDOM<br>2 HAS MORE THAN ONE SEX PARTNER<br>3 HAD BLOOD TRANSFUSION/INJECTION<br>4 HAS NOT BEEN CIRCUMCISED<br>5 HAS AN HIV POSITIVE PARTNER<br>6 OTHER________________<br>(SPECIFY)	
13	Have you ever taken an HIV test before?	1 YES → **B15**<br>2 NO	

14	What is the main reason why you have never tested? (choose only one)	1 DO NOT KNOW WHERE TO GO<br>2 TOO FAR<br>1 TOO TIME CONSUMING<br>2 EMBARRASSMENT<br>3 I KNOW I AM NEGATIVE<br>4 LACK OF PRIVACY/AFRAID TO BE SEEN<br>7 I AM AFRAID TO KNOW THE RESULT<br>7 I AM AFRAID TO BE TREATED BADLY<br><br>**SKIP TO B21 AFTER THIS**	
15	What was the result of the most recent HIV test that you took?	0 HIV-NEGATIVE<br>1 HIV-POSITIVE<br><br>98 DON'T KNOW<br>99 REFUSE TO ANSWER	
16	Approximately how many times have you been tested for HIV <u>in the past 12 months</u>?	NUMBER OF TESTS (2 DIGITS)<br><br>98 DON'T KNOW	
17	When was the most recent time you took a test for HIV?	1 LESS THAN 6 MONTHS AGO<br>2 ABOUT 6-12 MONTHS AGO<br>3 12-24 MONTHS AGO<br>4 MORE THAN 2 YEARS AGO<br><br>98 DON'T KNOW<br>99 REFUSED TO ANSWER	
18	What was the location where you had your most recent HIV test?	1 PUBLIC HEALTH FACILITY<br>2 PRIVATE CLINIC<br>3 COMMUNITY HEALTH CAMPAIGN<br>4 MOBILE CLINIC<br>5 AT MY HOME WITH A HEALTH COUNSELLOR<br>6 AT MY HOME USING A SELF-TEST<br>7 OTHER (SPECIFY)<br><br>99 REFUSED TO ANSWER	
19	Did you tell your primary partner the results of the HIV test?	1 YES<br>2 NO<br>3 No primary partner →**B22**<br><br>99 REFUSED TO ANSWER	
20	Did you tell anyone other than your primary partner the results of the HIV test?	1 YES<br>2 NO<br><br>99 REFUSED TO ANSWER	
21	Do you know your primary partner's HIV status?	1 YES<br>2 NO	
22	Have you heard about antiretroviral therapy (ART), the medicines for treating HIV?	1 YES<br>2 NO →**B24**<br><br>98 DON'T KNOW	
23	Do you know any persons who are currently taking ART medicines?	1 YES<br>2 NO<br><br>98 DON'T KNOW	

READ TO RESPONDENT: NOW I HAVE SOME QUESTIONS ABOUT SEXUAL ACTIVITY, IN ORDER TO GAIN A BETTER UNDERSTANDING OF SOME IMPORTANT ASPECTS OF YOUR LIFE.			
24	How old were you when you had sexual intercourse for the very first time?	AGE IN YEARS<br><br>0 IF NEVER HAD SEXUAL INTERCOURSE →**C1**<br><br>97 IF DON'T KNOW AGE OF FIRST SEX, BUT KNOW IT WAS WHEN FIRST STARTED LIVING WITH FIRST (SPOUSE OR PARTNER)<br><br>98 DON'T KNOW<br>99 REFUSED TO ANSWER	
25	When was the <u>most recent</u> time you had sexual intercourse?	1 Past 7 days<br>2 Past month<br>3 Past 3 months<br>4 Past 6 months<br>5 Past 1 year<br>6 More than 1 year ago<br><br>99 REFUSED TO ANSWER	
26	Did you use a condom during the most recent time you had sexual intercourse?	1 YES<br>2 NO<br><br>99 REFUSED TO ANSWER	
27	Was the person with whom you had sexual intercourse most recently your primary sexual partner?	1 YES<br>2 NO<br><br>99 REFUSED TO ANSWER	
28	Have you had sexual intercourse with any persons other than your primary sexual partner in the past 12 months?	1 YES<br>2 NO →**C1**<br><br>99 REFUSED TO ANSWER	
29	In total, with how many different persons have you had sexual intercourse in the past <u>12 months</u>?<br><br>IF NON-NUMERIC RESPONSE, PROBE TO GET AN ESTIMATE	NUMBER OF PARTNERS<br><br>98 DON'T KNOW<br>99 REFUSED TO ANSWER<br>IF NUMBER OF PARTNERS IS 97 OR MORE, WRITE '97'	
30	In the past 12 months, did you ever pay or receive gifts from anyone (cash or other items) in exchange for having sexual intercourse?	1 YES<br>2 NO →**C1**	
31	The last time you paid or received gifts from someone in exchange for having sexual intercourse, was a condom used?	1 YES<br>2 NO → **C1**	

Section C. Additional HIV testing and service delivery questions			
NO.	QUESTIONS	CODING CATEGORIES	RESPONSE
1	How likely would you say you are to go for an HIV test again sometime in the next 3 months?<br><br>READ ALL ANSWER CHOICES	1 VERY LIKELY<br>2 SOMEWHAT LIKELY<br>3 VERY UNLIKELY<br><br>98 DON'T KNOW	
2	How likely would you say you are to go for an HIV test again sometime in the next 12 months?<br><br>READ ALL ANSWER CHOICES	1 VERY LIKELY<br>2 SOMEWHAT LIKELY<br>3 VERY UNLIKELY<br><br>98 DON'T KNOW	
3	What concerns do you have about getting an HIV test?<br><br>RECORD ALL MENTIONED; DO NOT READ CHOICES TO RESPONDENT	1 COST OF TRAVEL TO PLACE WHERE HIV TESTING IS PERFORMED<br>2 COST OF MISSING WORK<br>3 FEARFUL OF LEARNING HIV STATUS<br>4 FEARFUL OF SOMEONE ELSE LEARNING YOUR HIV STATUS<br>5 PARTNER IS OPPOSED<br>6 NO CONCERNS<br>7 OTHER:______________ (SPECIFY)	
4	Have you ever heard about HIV self-tests, which allow you to test yourself for HIV at home using a swab of your gums?	1 YES<br>2 NO<br><br>98 DON'T KNOW	
5	If you received a kit that allowed you to test yourself for HIV that is easy to use and is as accurate as tests at the health facility, how likely would you be to use the test?	1 VERY LIKELY<br>2 SOMEWHAT LIKELY<br>3 NOT VERY LIKELY<br><br>98 DON'T KNOW	

Section D. Loss aversion questions			
NO.	QUESTIONS	CODING CATEGORIES	RESPONSE
1	Imagine you are invited to play a game where you have the chance to win money but could also lose your own money. In the game, you have a 50% chance of winning 5,000 Shillings and 50% chance of losing 1,000 Shillings. Would you play the game?	1 YES<br>2 NO →D5<br><br>98 DON'T KNOW	
2	Now suppose you have a 50% chance of winning 5,000 Shillings and 50% chance of losing 2,500 Shillings. Would you play the game?	1 YES<br>2 NO →D5<br><br>98 DON'T KNOW	
3	Now suppose you have a 50% chance of winning 5,000 Shillings and 50% chance of losing 5,000 Shillings. Would you play the game?	1 YES<br>2 NO →D5<br><br>98 DON'T KNOW	
4	Now suppose you have a 50% chance of winning 5,000 Shillings and 50% chance of losing 6,000 Shillings. Would you play the game?	1 YES<br>2 NO<br><br>98 DON'T KNOW	
5	Suppose you are a farmer and decided to buy fertilizer for growing your crops. You used the fertilizer then a big storm destroyed some of your crops. How upset would you feel?	1 A LITTLE UPSET<br>2 SOMEWHAT UPSET<br>3 VERY UPSET<br><br>98 DON'T KNOW	
6	Suppose you are a farmer and decided to buy fertilizer for growing your crops. You used the fertilized and it was a good planting season. You grew more crops than you expected. How happy would you feel?	1 A LITTLE HAPPY<br>2 SOMEWHAT HAPPY<br>3 VERY HAPPY<br><br>98 DON'T KNOW	

Section E: RANDOMIZATION

INTERVIEWER: GIVE THE RESPONDENT A SCRATCH CARD AND ASK PARTICIPANT TO SCRATCH OFF THE SHADED AREA

NO.	QUESTIONS	CODING CATEGORIES	RESPONSE
1	What is the randomization number?		
2	INTERVIEWER: Re-enter the randomization number	*Verify this matches previous number*	
3	What is the study group that is revealed?	1 CONTROL (No incentive) →**E7**<br>2 NO DEPOSIT/GAIN →**E7**<br>3 SMALL DEPOSIT/LOSS-1<br>4 LARGE DEPOSIT/LOSS-2	
4	Did the participant accept or decline the deposit?	1 Accept<br>2 Decline →**E6**	
5	How much more would the participant be willing to deposit?	*Write amount (UGX)*<br>**SKIP TO SECTION F**	
6	Reason for declining the deposit.	**SKIP TO SECTION F**	
7	Would the participant be willing to deposit money that they could receive, with interest, after their next HIV test?	1 Yes<br>2 No	

Section F: Interviewer's Observations

TO BE ANSWERED BY INTERVIEWER AFTER INTERVIEW IS FINISHED

NO.	QUESTIONS	CODING CATEGORIES
1	Interviewer: Use this field to record any special comments/info/problems	
2	Re-scan/re-write bracelet ID	

**END TIME OF INTERVIEW**<br>(HH:MM:SS)	
