# Supplementary material for: A pilot randomized trial of incentive strategies to promote HIV retesting in rural Uganda
Source: PLoS One. 2020 May 29;15(5):e0233600. doi: 10.1371/journal.pone.0233600 (PMC7259772; doi:10.1371/journal.pone.0233600)
Supplement: Attachment — Submitted filename: Response to Reviewers Retest Pilot v31Jan2020.docx [file pone.0233600.s004.docx]

**Response to Reviewers & Editor**

**Title**: A pilot randomized trial of incentive strategies to promote HIV retesting

**Manuscript**: PONE-D-19-27665

**Comments from the Editor:**

When submitting your revision, we need you to address these additional requirements.

*1. Please ensure that your manuscript meets PLOS ONE's style requirements, including those for file naming. The PLOS ONE style templates can be found at*

[*http://www.journals.plos.org/plosone/s/file?id=wjVg/PLOSOne_formatting_sample_main_body.pdf*](https://urldefense.proofpoint.com/v2/url?u=http-3A__www.journals.plos.org_plosone_s_file-3Fid-3DwjVg_PLOSOne-5Fformatting-5Fsample-5Fmain-5Fbody.pdf&d=DwQGaQ&c=iORugZls2LlYyCAZRB3XLg&r=T1DDCoJhuKu3mKWJycC5Ga4tV8FWp5Edo1PzXzcA8sw&m=EQ71OYMLnWnMyf-zl207VqbCFndOwm0xBZs6Ywq4oE8&s=YG7_P_wBCxBCe8LMbyeQmDm438dRx8YuPsWy0qTfbUA&e=) *and* [*http://www.journals.plos.org/plosone/s/file?id=ba62/PLOSOne_formatting_sample_title_authors_affiliations.pdf*](https://urldefense.proofpoint.com/v2/url?u=http-3A__www.journals.plos.org_plosone_s_file-3Fid-3Dba62_PLOSOne-5Fformatting-5Fsample-5Ftitle-5Fauthors-5Faffiliations.pdf&d=DwQGaQ&c=iORugZls2LlYyCAZRB3XLg&r=T1DDCoJhuKu3mKWJycC5Ga4tV8FWp5Edo1PzXzcA8sw&m=EQ71OYMLnWnMyf-zl207VqbCFndOwm0xBZs6Ywq4oE8&s=RdEyZmSL1fqnzzfbBOUl-zk5LZXTuh7w_1QvD2U2yc8&e=)

**Response:** We have ensured that our manuscript meets PLOS ONE’s style requirements, including those for file naming.

*2. Please provide additional details regarding participant consent. In the ethics statement in the Methods and online submission information, please ensure that you have specified whether consent was suitably informed.*

**Response:** We now provide additional detail regarding participant consent in the Methods (see lines 169-170). All participants provided written informed consent in their preferred language (English or Runyankole, the local language in the region).

*3. We note that you have stated that you will provide repository information for your data at acceptance. Should your manuscript be accepted for publication, we will hold it until you provide the relevant accession numbers or DOIs necessary to access your data. If you wish to make changes to your Data Availability statement, please describe these changes in your cover letter and we will update your Data Availability statement to reflect the information you provide.*

**Response:** Noted – thank you. We will provide the study dataset (Supporting Information 1: S1, noted on line 307) should our manuscript be accepted for publication.

*4. Please include captions for your Supporting Information files at the end of your manuscript, and update any in-text citations to match accordingly. Please see our Supporting Information guidelines for more information:*[*http://journals.plos.org/plosone/s/supporting-information*](https://urldefense.proofpoint.com/v2/url?u=http-3A__journals.plos.org_plosone_s_supporting-2Dinformation&d=DwMGaQ&c=iORugZls2LlYyCAZRB3XLg&r=T1DDCoJhuKu3mKWJycC5Ga4tV8FWp5Edo1PzXzcA8sw&m=EQ71OYMLnWnMyf-zl207VqbCFndOwm0xBZs6Ywq4oE8&s=mnG9jIbN7pF-2ExGpKL-_1Ddn942s8oX4FilAmjeWkw&e=)

**Response:** We now provide captions for our Supporting Information files at the end of our manuscript (see lines 306-309).

**Response to Reviewers’ Comments**

**Reviewer #1**

*1) Interesting study to incentivize retesting of negative individuals vs. incentivizing staying HIV negative. Considering the WHO 2015 Consolidated guidelines on HIV testing services and the upcoming update being released in December 2019 recommends that for most people who test HIV negative additional retesting to rule out being in the window period is not necessary (after 3 months). In addition, the WHO guidelines recommends retesting of individuals with ongoing high risk of HIV infection, not all key populations require retesting every 3 months. It would be helpful for the authors to address the above in the introduction and discussion.*

**Response**: We now address, in both the Introduction and Discussion, retesting at 3-month intervals and annually (see Introduction, lines 61-66, and Discussion lines 284-292). We reference the WHO Consolidated HIV Testing Guidelines, and now include reference to the November 2019 update as well, which – though the update recommends annual retesting for all sexually active individuals in high HIV burden settings (such as Southwestern Uganda, where our pilot took place) – also notes that frequent retesting for HIV (i.e. every 3-6 months), “may be warranted based on individual risks and as part of broader HIV prevention interventions.” Uganda Ministry of Health guidelines at the time of this pilot (and currently) recommended retesting for HIV every 3 months in key populations – distinct from WHO guidelines, which we now address in the Introduction (see lines 61-66). In this pilot study, we worked with local health officials and community representatives in a generalized epidemic setting (Southwestern Uganda) to identify specific venues frequented by adults that the health officials/community leaders considered to be high-risk for HIV in their community. The HIV positivity of adults tested during recruitment (15%) is more than double that of the population HIV prevalence in the region (7%), suggesting that the input of local health officials/community leaders was accurately identifying high-risk venues.

*2) The methods of offering incentives is good; however, please address why incentivizing retesting of negative individuals vs. behavior change to remain negative.*

**Response**: The reviewer correctly notes that various behaviors can be incentivized to prevent HIV, including a variety of prevention behaviors such as voluntary medical male circumcision, condom use, HIV retesting or other behaviors. However, many behaviors (condom use, changes in sexual behavior) cannot ethically or practically be observed or verified for the use of conditional financial incentives or deposit contracts. We therefore focused our efforts on a behavior we could verify (HIV retesting) and that can also act as a platform for ongoing counseling and linkage to other emerging combination prevention services over time.

*3) Also address why incentivizing retesting of HIV negative individuals in a resource limited setting that is focusing more on targeted HIV testing services (e.g. partner notification services, risk screening tools in outpatient settings) with linkages to treatment and/or prevention services.*

**Response**: Retesting of high-risk HIV negative individuals is a form of targeted HIV testing services, as reflected in Ugandan Ministry of Health guidelines for every 3-6 month retesting for key populations, such as the adults we recruited into our study. At the time of our pilot, pre-exposure prophylaxis (PrEP) was not available in government clinics; however, as PrEP becomes increasingly available, retesting at regular intervals (typically every 3 months in PrEP programs) will have even greater relevance among adults accessing PrEP services.

*4) Please address how incentivizing retesting of the study participants is better than current practice in clinics or other HIV testing locations that offer HIV testing services? Please include references that show that there is a need to incentivize retesting of individuals who are HIV negative. There is a lot of peer reviewed literature that shows most HIV negative individuals (with or without ongoing risk) do come back for retesting.*

**Response**: The primary objective of our pilot trial was not to directly compare the effectiveness of incentivizing HIV retesting vs. counseling participants to retest (current practice), and as such we cannot directly address how incentivizing retesting is better than current practice with our study data. However, regarding the need for interventions to promote retesting (the crux of the Reviewer’s insightful comment), peer-reviewed literature on retesting is fairly limited – but the several studies published on this topic have documented low rates of retesting at a frequency of at least annually (if not more frequently, such as every 3-6 months, as recommended for high-risk individuals: see response to first Reviewer #1 comment). For example, in a 2011 study that examined mobile testing outreach in South Africa, although 71% of adults tested reported *any* prior HIV test, only 37% reported testing within the prior 12 months (reference: Kranzer et al, *High Prevalence of Self-Reported Undiagnosed HIV despite High Coverage of HIV Testing: A Cross-Sectional Population Based Sero-Survey in South Africa*. PLoS One, 2011). Similarly, in a 2013 study from Tanzania evaluating individuals retesting for HIV over three time periods from 2003-10, only 11% of adults participated in retesting (reference: Cawley, C. et al. *Low rates of repeat HIV testing despite increased availability of antiretroviral therapy in rural Tanzania: findings from 2003–2010.* PloS ONE 8, e62212, 2013), and in a 2013 study, Regan et al found only 26% of adults reporting retesting in two South African hospitals (reference: Regan et al, *Factors Associated with Self-Reported Repeat HIV Testing after a Negative Result in Durban, South Africa*, PLoS One, 2013). Based on our prior work in Southwestern Uganda that sought to recruit all adult men across a community for HIV testing, though any prior HIV testing was common (78% of men), *less than half* reported testing within the past year (Chamie, et al, AIDS, 2018). These published data highlight low rates of HIV retesting, falling well below WHO recommendations of annual retesting for all adults, let alone more frequent testing of high-risk adults. Lastly, in a recent systematic review of interventions to promote retesting, the authors noted that, “evidence-based interventions that promote frequent and repeat testing more than once a year among individuals at high risk for HIV remain sparse,” (reference: Paschen-Wolff, et al, *A Systematic Review of Interventions that Promote Frequent HIV Testing*, AIDS and Behavior, 2019), emphasizing the need to better study interventions to promote retesting.

We now review this literature in greater detail in the revised Introduction section (see lines 67-79, and 80-92).

**Reviewer #2:**

*General Comments:

Background: The study authors outline how the use of financial incentives in the role of HIV re-testing have yet to be studied. While this might be the case, I think the authors could make a stronger case for why the use of financial incentives for retesting would expect to be any different than the use of financial incentives for HIV testing in general? For example, do the authors expect a different outcome than what research has shown with financial incentives for HIV testing? Please clarify.*

**Response**: We have now clarified in greater detail why retesting for HIV after a negative test is likely to be a distinct behavior from incentivizing HIV testing in general (see Introduction, lines 80-92). Motivation to retest for HIV after a negative test may be lower than testing for HIV in general, given studies that have shown perceptions that retesting is unnecessary (e.g. Orne-Gliemann, J., et al. (2016). "Community perceptions of repeat HIV-testing: experiences of the ANRS 12249 Treatment as Prevention trial in rural South Africa." AIDS care 28 Suppl 3: 14-23).

*Background: This manuscript would benefit from some additional references, particularly in the Background section to provide a stronger case for how the use of financial incentives for HIV retesting would be beneficial for the field. For example, what is the prevalence and/or incidence of HIV infection in Uganda? How often do people get retested in Uganda? If the study authors provided some brief background information about HIV and HIV retesting uptake in Uganda this might also increase clarity for the international reader as to why innovative interventions are needed in this country/region.*

**Response**: We now provide additional references and information in our Introduction section to offer more background information about the need for interventions to promote retesting for HIV, and about HIV and retesting uptake among high-risk adults available from published studies in sub-Saharan Africa, including Uganda. (See Introduction, lines 67-79, and 80-92).

*Discussion: Please provide some detail as to how the study findings are comparable to other studies that use financial incentives in low-and-middle income countries in the HIV field.*

**Response**: We now include greater detail on how our study findings are comparable to other studies that have used incentives for HIV testing in low- and middle-income settings, including reference to a systematic review of incentives for HIV testing (see Discussion, lines 251-270).

*Discussion: Please provide a brief statement how the findings from this study could inform future research in the field, and also, remaining knowledge gaps and areas for future research.*

**Response**: We now provide a brief statement about how the findings from this study could inform future research in the field and have provide knowledge gaps remain as a basis for areas of future research (see Discussion, lines 265-270).

*Specific Comments:

Abstract: In the Results section, the authors have listed number/percentage to display one set of findings but then only percentages for the remaining findings. Please use number/percentage to be consistent.*

**Response**: We now provide number/percentages throughout the Results section of the Abstract for consistency.

*Introduction: “Economic incentives have been shown to promote a number of behaviours…” This paragraph is not entirely clear. For example, is ‘counseling alone’ the standard of care?*

**Response**: We have revised this paragraph (see Introduction, lines 80-92) for greater clarity, including the standard of care, which is counseling alone in this setting.

*Please rephrase this sentence: “the costs of a given behavior are immediate but the gains may not be realized until later…” (could the authors please provide an example?). Also, with this sentence: “insights from behavioural economic theory suggest that individuals display loss aversion…” Perhaps the authors provide an example to increase clarity of “loss aversion”?*

**Response**: We have rephrased these sentences highlighted by the reviewer, providing examples for the phrase discussing “costs of a given behavior”, as well as an example to clarify loss aversion (see Introduction, lines 80-84, and lines 93-99).

*Introduction: “A standard approach to implementing…” Please provide some references here to increase clarity.*

**Response**: We now provide references for this section (Introduction, lines 93-95).

*Introduction: “Individuals may also be aware of a tendency towards present-biased…” Please modify this sentence to increase clarity.*

**Response**: We have revised this sentence in an effort to increase clarity (lines 99-110).

*Introduction: What is “Leverage loss aversion”? Please clarify.*

**Response**: We have revised this sentence in an effort to increase clarity (lines 95-99).

*Methods: How long did the authors give the participants to be re-tested? Did participants have to come back at exactly 3 months post study enrollment for re-testing? Please add some study details to increase clarity.*

**Response**: As we note in the Methods (please see Methods, lines 155-157) participants did not have to return for retesting at exactly 3 months post-enrollment but were instead provided an interval of 2-3 months to return to the clinic for retesting.

*Methods: “considered at increased risk of HIV, due to drinking, sex work” What does “due to drinking” mean in this case? Please remove or modify to increase clarity.*

**Response**: We now have modified this phrase for further clarity (see Methods, lines 126-128). The phrase now states, “due to drinking at bars associated with transactional sex.”

*Methods: What was the questionnaire that participants were asked to complete? Did the questionnaire only enquire about participant demographics or also measure ‘feasibility’ and ‘acceptability’? How many questions were in the questionnaire? Who developed the questionnaire? Who administered the questionnaire? Was it self-administered?*

**Response**: The questionnaire was developed by the investigators, administered by study staff, and sought to gather information on both demographics, socioeconomic status, HIV risk behavior, prior HIV testing behavior and testing preferences. There were 62 questions in the questionnaire. These details are now provided in the Methods, (see lines 143-145).

*Methods: Please specifically define ‘feasibility’ and ‘acceptability’ and provide a reference, if possible. Also, please define how these outcomes are measured. In the Discussion section, it states, “were feasible and had high acceptability”, however, it is not clear how these outcomes were measured (and what could be defined as ‘high acceptability’).*

**Response**: We now define acceptability in our methods (see lines 160-162. We pre-specified our definition of acceptability and feasibility in our study protocol (included with our PLoS One submission). Feasibility and how we measured these outcomes are clearly defined in our Methods as well (see lines 162-163). As this was a pilot trial, we did not pre-specify the definition of “high” acceptability, but we now clarify in our Discussion (line 221) that when we indicate “high acceptability”, this refers to the finding that 95% of eligible participants agreed to proceed with enrollment and continued with trial participation after randomization.

*Methods: Please define “standard, gain framed cash incentive” to increase clarity. Similarly, what is a “gain framed” incentive?*

**Response**: A “gain-framed” incentive is a simple financial reward (or bonus) provided for doing a given, desired behavior. We now clarify this phrase (see line 150).

*Methods: Where did participants need to go to receive retesting? At one clinic site? At several clinic sites?*

**Response**: Participants needed to return to the same, single clinic site to receive retesting. We now clarify this point in the Methods (see lines 159-160).

*Methods: What is standard of care in this case? i.e. “no incentive (control)”*

**Response**: The standard of care is counseling to promote retesting without the use of an incentive (i.e. what we provided in our control arm: no incentive, but counseling) – and this standard is now clarified in our Introduction (see lines 90-91).

*Methods: “we determined sample size…in feasibility of commitment contracts in approximately 70 adults rather than using power calculations…” Please consider modifying this section to increase clarity. It is not clear how the study authors determined a sample size of 70 to measure feasibility. Further, was the sample size determined to only measure feasibility or also acceptability? At this point, the study authors could state the minimum number of participants needed in each condition to be powered to detect statistical significant differences between conditions. The study authors could also emphasise in the Results section that the study was not powered to detect statistical significance between conditions, and in the Discussion section, mention that this is an area for future research (would be powered in future trial).*

**Response**: We did not use power calculations to determine a sample size, and we feel that presenting the sample sizes needed to detect statistical differences between arms (which is a total sample size of 525 participants in our subsequent 3-arm trial to promote retesting) would introduce more confusion than clarity. We now emphasize in our Results section that the study was not powered to detect statistically significant differences in retesting between arms (see lines 214-215) and mention that this is an area for future research in the Discussion section (see lines 224-225).

*Methods: Could the study authors please provide greater details as to the venues in which the participants were recruited from? Is there any indication of how many venues the participants were recruited from? Some detail is mentioned in the Results section but it is not very clear, e.g. a clinic waiting room (primary care clinic waiting room?), bars (what kind of bars? How many?). For example, how many transport hubs? How many trading centers? Additionally, what are the “other sites”?*

**Response**: We now include greater detail on the venues from which participants were recruited in the Methods section (see page 7, lines 127-132). We also now include greater detail on the venues in our Results section, including clarification as to what the “other sites” were and the number of venues (see page 10, lines 179-182).

*Results: What is the “Figure: Pilot trial CONSORT diagram”? (p.9)*

**Response**: This Figure was submitted with the manuscript, and is a CONSORT diagram, now referenced, that is recommended to use to provide detail on recruitment, enrollment and retention of participants in pilot trials. We have elaborated on this in the description of the Figure (see lines 201-202).

*Results: It would be helpful if Table 1 was reformatted as it is currently challenging to read when the findings take the space of two rows.*

**Response**: The formatting is based on PLoS One requirements to have double spacing of tables and is not how the table will appear if accepted for publication by PLoS One. Ultimately, if the manuscript is accepted, we will request that the table have findings on a single row.

*Discussion: “individuals’ ability to make deposits may be limited by poverty and other circumstances.” Please provide an example of “other circumstances”. Also, are there any ethical issues to administering commitment contracts in low-and-middle income countries?*

**Response**: We have removed the phrase, “other circumstances,” as we feel poverty is the primary driver of one’s ability to make a deposit (see line 232). As commitment contracts are voluntary, and suggested deposits are considered low, we do not believe there are any ethical issues to administering commitment contracts.

*Discussion: “This approach also addressed a limitation observed in commitment contract programs in high-and middle-income countries…” Please expand on how this approach addressed this limitation?*

**Response**: We now expand on how this approach addressed this limitation (see Discussion, lines 236-238).

*Discussion: “was not powered to detect differences in retesting uptake…” Differences by study condition? Differences by participant characteristics? Please elaborate.*

**Response**: We now clarify this phrase, as it was used to indicate detection of differences by study condition (see lines 273).

*Discussion: “In future studies with more than one retesting opportunity, we suspect retesting might decline over time when incentives are not offered.” Please provide a reference here. Is this based on prior research?*

**Response**: This suspicion that retesting might decline over time is based on the observation that with healthy behaviors that require repeated/continued engagement (such as smoking cessation or exercise), the withdrawal of incentives can lead to a decline in healthy behavior from the levels of behavior achieved with an incentive. We also now include an example and a reference for this tendency observed in prior research in the manuscript (see line 279).

*Discussion: “Our pilot trial was designed to test acceptability and feasibility…” Is this paragraph a study limitations paragraph? If yes, please state more directly. For example, “These observations, along with the high prevalence of HIV among adults screened (15%...)…” It is not clear if this was stated as a study limitation or strength. Please clarify. Also, the study authors could mention if there were any study biases and how the study authors tried to respond or account for these.*

**Response**: We now directly state that this paragraph is a study limitations paragraph, and enumerate our limitations (“First”, “Second”, “Third”, etc.), to distinguish them from the strengths we highlight in response to these limitations (see lines 271-296).

*Conclusion: “As reported rates of “ever” having tested…” As the sentence is currently structured, it seems like the study authors are stating that retesting is of “greater priority” than first-time testing. Is this what the study authors meant? Or that “retesting” is also of importance? Please clarify.*

**Response**: We are not stating that retesting is of greater priority than first-time testing in general, but rather making the point that as rates of “ever testing” reach very high levels, the relative importance of retesting increases. We now clarify this point (see lines 299-300).
