# Supplementary material for: A pilot randomized trial of incentive strategies to promote HIV retesting in rural Uganda
Source: PLoS One. 2020 May 29;15(5):e0233600. doi: 10.1371/journal.pone.0233600 (PMC7259772; doi:10.1371/journal.pone.0233600)
Supplement: Attachment — Submitted filename: Response to Reviewers Retest Pilot v24Mar2020.docx [file pone.0233600.s005.docx]

**Response to Reviewers & Editor**

**Title**: A pilot randomized trial of incentive strategies to promote HIV retesting

**Manuscript**: PONE-D-19-27665

**Response to Reviewers’ Comments**

**Reviewer #1**

*General Comments:*

*It is technically sound and all comments were addressed. However, incentivizing retesting of negative individuals vs. prevention to remain negative in resource limited settings is not sustainable and somewhat conflicts the updated 2019 consolidated guidelines recommendations on targeted testing in changing epidemics.*

**Response**: We are pleased that Reviewer 1 finds all of the comments addressed and the manuscript technically sound. The use of incentives are endorsed by the WHO 2019 Consolidated guidelines, and could be used to increase retesting that is targeted to very high-risk people who test negative for HIV, but based on risk behavior, merit retesting 3-6 months in the future – a recommendation in line with WHO guidelines. Furthermore, deposit contracts are potentially cost saving compared to standard incentives, as they leverage voluntary deposits by adults who wish to participate in them. However, our manuscript is not designed to evaluate or test the sustainability of this approach, but rather to understand the feasibility and acceptability of incentive strategies to promote HIV retesting. We agree that further research is needed to address the question of cost-effectiveness and sustainability.

**Reviewer #2:**

*General Comments:*

*The study authors have made some substantial changes to this manuscript, which has helped to increase clarity. I have provided a few more suggested modifications for your consideration:*

*Specific Comments:

Title: The title is a bit too concise. Please consider adding the country/location of the study and/or the study population group. For example, “to promote HIV restesting among …. In Uganda”*

**Response**: We have revised the title to include the study location as rural Uganda.

*Abstract: “Retesting for HIV is critical to identifying newly-infected persons and reinforcing prevention…” The ‘reinforcing’ term is unclear. Do the authors mean reinforcing prevention efforts? Prevention messaging? Please modify. Page 4 of 6*

**Response**: We have revised this sentence to clarify that HIV retesting can reinforce prevention efforts among at-risk adults.

*Abstract: “Incentives can increase one-time testing…” Based on? Perhaps better to put ‘may increase’?*

**Response**: This statement in the abstract is based on multiple studies, including trials and a systematic review, that have demonstrated that incentives can increase one-time HIV testing. We provide these references in our Introduction section (page 6, lines 81-85).

*Entire Manuscript: As much as possible, it is better to use ‘HIV retesting’ instead of ‘retesting’ as a firm reminder to the reader that the authors are referring to retesting of HIV. For example, Pg. 9 “the deposits would be lost if they did not return for HIV retesting …”. Also, Pg.14, “clear understanding of the benefits of HIV retesting …”*

**Response**: We have revised the manuscript to emphasize that our aim and outcome was increase in HIV retesting. These clarifications include the reviewer’s specific suggestions to add “HIV” to the term “retesting” on pages 9 and 14, as well as throughout the manuscript.

*Abstract: “to promote retesting three months later…” Do the authors mean, three months post-study enrolment? Please clarify.*

**Response**: We have revised this sentence to clarify that our outcome was HIV retesting at three months after study enrollment.

*Abstract: “Contracts sought to promote retesting by leveraging …” The study authors explain this sentence in the manuscript, so fine to leave there, but it is a bit confusing in the Abstract. Could remove.*

**Response**: We recognize that this term is not fully defined in the abstract, but it is a well-known behavioral economic term that is well-defined in our manuscript, and key to how our deposit intervention works, so we have chosen to leave it in the manuscript.

*Abstract: “(proportion making deposits)” – Perhaps, ‘proportion of participants making deposits’?*

**Response**: We have revised this sentence to clarify that feasibility was assessed as the proportion of participants who chose to make a deposit.

*Abstract: “Overall, 93 (76%) participants retested” – Please consider adding “were retested for HIV” – Again, making clear to the reader that we are speaking to retesting of HIV.*

**Response**: We have revised this sentence to clarify that our outcome was HIV retesting uptake.

*Introduction: The first section of the Introduction is verbose and hard to understand. Please modify. For example, what are combination prevention approaches?*

**Response**: We now provide some examples of combination HIV prevention approaches to provide greater clarity to the first paragraph of the Introduction.

*Introduction: “Retesting of high-risk, HIV-uninfected persons is critical to…” Please add a reference after this point.*

**Response**: We now provide references for this sentence.

*Introduction: “Retesting also offer an opportunity to …” Please add a reference after this point.*

**Response**: We now provide a reference for this point.

*Introduction: “may be perceived as low, particularly if access” Access to what? A clinic? Please clarify.*

**Response**: This sentence has been revised to specify that access to testing and transportation to clinics may be costly for some individuals.

*Introduction: “incentives can be made more powerful”. I am not sure that ‘powerful’ is the right word here. Do the authors mean ‘effective’ or ‘impactful’?*

**Response**: This sentence has been revised to clarify that the use of loss aversion principles can make incentives more effective.

*Introduction: “Recent studies have indicated that …” Please add the references to the ‘recent studies’ here.*

**Response**: We now provide references for this sentence.

*Introduction: “Very few incentive-based approaches…” Please add references here to support this statement.*

**Response**: This phrase is based on our own extensive review of the literature, and does not have a specific reference, as currently there is no systematic review of incentive-based approaches that leverage loss aversion. We have revised this sentence to clarify that, “to our knowledge, no incentive-based approaches in sub-Saharan Africa have attempted to leverage loss aversion for behavior change through commitment contracts.”

*Introduction: “We assessed the feasibility and acceptability …” This statement could be made stronger by stating, “The aim of this study is to…”*

**Response**: This sentence has been revised, based on the reviewer’s suggestion to strengthen the message and highlight the study aim.

*Methods: “due to drinking at bars associated with transactional sex…” This sentence is unclear. Do the authors mean that the “adults considered at increased risk of HIV due to attendance at … and behaviours in sex work …?” Please modify.*

**Response**: This sentence has been revised to clarify that individuals who attended bars and sites of sex work were considered to be at increased risk of HIV-infection and were specifically recruited for this study.

*Methods: “rapid HIV antibody testing”. Could the study authors please list the type of test that was used (e.g. brand name, etc.).*

**Response**: The study performed HIV antibody testing using the Alere Determine^TM^ HIV-1/2 Ag/Ab Combo, followed by the Chembio HIV 1/2 STAT-PAK® and SD Bioline HIV-1/2 assays for confirmation of Determine-positive results. This information has been added to the Methods section.

*Results: “HIV positivity varied by recruitment site …” Was this variance expected? If no, please list this more directly in the Limitations section. For example, following the sentence, “Possibly suggesting that we did not reach a high-risk population group through our selection of recruitment study sites …”*

**Response**: This variance was expected.

*Results: Please list as ‘Figure 1’ and ‘Table 1’.*

**Response**: The figure and table labels have been revised to read “Figure 1” and “Table 1” as suggested.

*Results: Figure 1 is quite helpful and should be included as part of the manuscript instead of supplementary material, if possible (i.e. it was not embedded in the manuscript like the Table).*

**Response**: We are pleased that Reviewer 2 finds Figure 1 helpful. Figure 1 will be included as part of the manuscript (it is not supplementary material). However, PlosOne requires that figures be uploaded as separate files and not be embedded in the manuscript.

*Discussion: “the optimal balance between deposit amounts that are acceptable versus of sufficient value to generate …” I know what the authors are trying to say, but I think it is the ‘versus’ that makes the statement a bit awkward. Could change to ‘that are acceptable and of sufficient value’. Page 5 of 6*

**Response**: This sentence has been revised to clarify that future studies may need to determine the optimal balance between deposit contract amounts that are acceptable and of sufficient value to generate loss aversion.

*Discussion: “other HIV-related behaviours and outcomes, such as voluntary medical male …” If the study authors keep the examples of VMMC and HIV viral suppression in this specific sentence, then references should be used. The authors could also remove these specific examples in this sentence, given that the examples are provided in the following sentence with references. It keeps the overall paragraph less repetitive. For example, “other HIV-related behaviours and outcomes. The effectiveness of these strategies when studied …Examples of effective strategies have included …”*

**Response**: Voluntary medical male circumcision and HIV viral suppression have been removed from the sentence indicated in this comment from the reviewer. These specific examples have been retained later in the same paragraph with references cited.

*Discussion: “decreasing engagement in risky sex among men…” This sentence is a bit unclear. Is this referring to men who have sex with men? Or more that men are engaging in high-risk sexual activities? Please clarify.*

**Response**: This sentence has been clarified to indicate that the study in question assessed high-risk sexual activity among men in Malawi.

*Discussion: “whether commitment contract participants…” This is a bit of lengthy adjective. Perhaps change to “whether participants who agree to commitment contracts…”*

**Response**: This sentence has been revised as suggested by the reviewer.

*Discussion: “Fourth, no seroconversions were observed…” Again, re-emphasise HIV. “Fourth, there were no HIV seroconversions observed in this study …”*

**Response**: This sentence has been revised to emphasize that the outcome being observed was HIV seroconversions.

*Discussion: “However, participants reported high-risk sexual behaviour…” The sexual behaviours of the participants were presented briefly in Table 1. Are there other components of the questionnaire that can be mentioned in paragraph 1 or 2 of the Results section or placed in Table 1? (but not to be mentioned in both places). The authors used a 62-item questionnaire, so there could be more Results presented from the questionnaire in either Table 1 or written in the Results section. The authors could also provide a copy of the 62-item questionnaire in the Supplementary information section.*

**Response**: The questions regarding sexual risk that we asked in our 62-item questionnaire were brief and are all included in Table 1. We now provide a copy of the 62-item questionnaire for inclusion in the Supplementary materials.

*It might be clearer if the study authors use ‘Table 1’ to highlight baseline characteristics of study participants (study demographics, sexual activity behaviours, etc.) and ‘Table 2’ to show study outcomes (e.g. ‘HIV retesting 2-3 months following randomisation’ ‘Deposit made to commitment contract’) because the title of Table 1 currently is baseline characteristics, and HIV retesting, for example, is not a baseline characteristic. There are also empty spaces in some parts of Table 1 and then ‘N/A’ in other sections, would be more helpful to be consistent.*

**Response**: We have opted to present a single table, as the retesting outcomes are brief (3 rows), and the title of Table 1 is “Baseline characteristics and HIV retesting uptake,” indicating that both outcomes are presented in this single table. We have added “N/A” to the empty spaces and thank the Reviewer for this suggestion.
