# Supplementary material for: A pilot randomized trial of incentive strategies to promote HIV retesting in rural Uganda
Source: PLoS One. 2020 May 29;15(5):e0233600. doi: 10.1371/journal.pone.0233600 (PMC7259772; doi:10.1371/journal.pone.0233600)
Supplement: Attachment — Submitted filename: Response to Reviewers Retest Pilot v21Apr2020.docx [file pone.0233600.s006.docx]

**Response to Reviewers & Editor**

**Title**: A pilot randomized trial of incentive strategies to promote HIV retesting

**Manuscript**: PONE-D-19-27665

**Response to Reviewers’ Comments**

**Reviewer #3**

*General Comments:*

*This is a very interesting study assessing the feasibility and acceptability of various incentive strategies, including commitment contracts that required a baseline deposit, to promote HIV retesting among at-risk adults in rural Uganda. Some minor comments considered important.*

*Specific Comments:*

*Although the methods sections is well detailed, the authors have not explicitly included a statistical analysis section. For example descriptive statistics presented as means(SD), median(IQR) and counts(%ages), also the authors have presented proportions with 95%CI using exact methods to obtain binomial confidence intervals. All this should be stated in the analysis section after methods or within the methods section.*

**Response**: We have revised the methods section to include a statistical analysis section (under a subheading within the Methods section), including additional detail about the statistical analyses performed for this study.

*There were a lot of baseline questionnaires collected but only a selection of variables have been presented in Table 1, for an example education, occupation, marital status, socio-economic status, as the readers gain more understanding of the characteristics of individuals who are identified as high risk for HIV infections. These categories could be grouped into sensible groups of course. These variables can added as an additional table in the manuscript.*

**Response**: We now include baseline education and marital status in Table 1, and socio-economic status is included as mean daily wage.

*Table 1: The variables “Daily wage (2017 US Dollars): mean”, please include SDs as well.*

**Response**: We now included standard deviations for these resulted variables.
